# Stepwise B-cell-dependent expansion of T helper clonotypes diversifies the T-cell response

**DOI:** 10.1038/ncomms10281

**Published:** 2016-01-05

**Authors:** Julia Merkenschlager, Mickaël J. Ploquin, Urszula Eksmond, Rakieb Andargachew, Georgina Thorborn, Andrew Filby, Marion Pepper, Brian Evavold, George Kassiotis

**Affiliations:** 1 Retroviral Immunology, The Francis Crick Institute, Mill Hill Laboratory, London NW7 1AA, UK; 2 Department of Microbiology and Immunology, Emory University School of Medicine, Atlanta, Georgia 30322, USA; 3 Department of Immunology, University of Washington, Seattle, Washington 98109-8059, USA; 4 Department of Medicine, Faculty of Medicine, Imperial College London, London W2 1PG, UK; 5 Present addresses: Institut Pasteur, Unité HIV Inflammation et Persistance, Paris 75015, France (M.J.P.); William Harvey Research Institute, Queen Mary University of London, London EC1M 6BQ, UK (G.T.); Faculty of Medical Sciences, Newcastle University, Newcastle upon Tyne NE1 7RU, UK (A.F.)

## Abstract

Antigen receptor diversity underpins adaptive immunity by providing the ground for clonal selection of lymphocytes with the appropriate antigen reactivity. Current models attribute T cell clonal selection during the immune response to T-cell receptor (TCR) affinity for either foreign or self peptides. Here, we report that clonal selection of CD4^+^ T cells is also extrinsically regulated by B cells. In response to viral infection, the antigen-specific TCR repertoire is progressively diversified by staggered clonotypic expansion, according to functional avidity, which correlates with self-reactivity. Clonal expansion of lower-avidity T-cell clonotypes depends on availability of MHC II-expressing B cells, in turn influenced by B-cell activation. B cells clonotypically diversify the CD4^+^ T-cell response also to vaccination or tumour challenge, revealing a common effect.

## Abstract (web summary)

During an immune response, CD4+ T cell repertoire is thought to increase in avidity at the expense of diversity. Here the authors show that B cells act as antigen-presenting cells to boost the development of low-avidity T cell clones, diversifying the T cell repertoire at late stages of the response.

## Graphical abstract

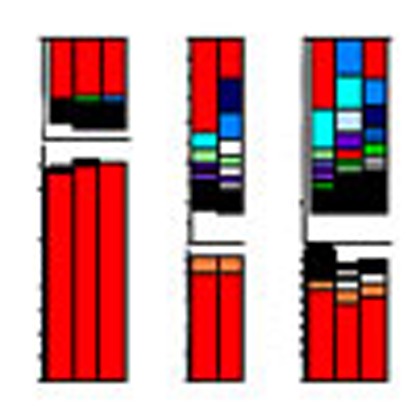

Adaptive immunity relies on clonal expansion and retention of antigen-specific lymphocytes bearing somatically generated and selected antigen receptors. Selection for lymphocytes with particular T-cell or B-cell receptors (TCRs and BCRs, respectively) occurs throughout the different stages of lymphocyte development, effector response and memory formation, based on thresholds of affinity for self or foreign antigen^1^,^2^,^3^,^4^. For example, the strength of TCR signalling is responsible for the selective advantage of high-affinity clonotypes that commonly dominate the peak CD4^+^ T-cell response^5^,^6^,^7^,^8^,^9^.

The selective forces driving the dominance of high-affinity CD4^+^ T cells during priming may continue to operate during memory formation. However, the relationship between primary expansion of a clonotype and memory formation is not always predictable. Indeed, the potential of distinct TCR clonotypes to form memory following LCMV infection does not correspond to the degree of their primary expansion^9^. Furthermore, comparison of two CD4^+^ T clones responding to *Listeria monocytogenes* infection revealed inverse behaviour during primary and secondary responses^10^, which is linked to avidity for self rather than foreign antigen, setting intrinsic thresholds for sensitivity and responsiveness^11^. The strength of self-reactivity, reflected in the expression levels of CD5, was also proposed as a clonotype-specific property directly related to the strength of TCR signalling in the response of several monoclonal or polyclonal CD4^+^ T cells to foreign antigen^12^. Moreover, individual CD4^+^ T cells bearing identical TCRs differ considerably in their ability to expand and differentiate in response to *L. monocytogenes* infection^13^, highlighting the influence of stochastic events, not linked to TCR affinity. In addition, the ratio of high- and low-affinity CD4^+^ T cells in response to vaccination is heavily influenced by the co-administered adjuvant^14^, the use of peptide instead of protein antigens^15^, the nature of the vaccine vector^16^ or simply by antigen dose^17^,^18^. Despite the potential of T-cell-extrinsic factors to influence the clonotypic composition of a T-cell response, their mechanism of action or amplitude are not yet fully understood.

Here, we used a well-characterized model to study the clonotypic evolution of the CD4^+^ T-cell response to retroviral infection. Inoculation of C57BL/6 (B6) mice with Friend virus (FV), a retroviral complex of Friend murine leukaemia virus (F-MLV) and spleen focus-forming virus (SFFV), causes protracted infection with several weeks of viral replication^19^,^20^. We have previously described a TCRβ-transgenic strain, which generates diverse CD4^+^ T-cell clonotypes with a range of functional avidities for the dominant H2-A^b^-restricted env_122–141_ epitope within the surface unit of the F-MLV *env* gene^8^,^21^. Our results revealed that the CD4^+^ T-cell clonotypic hierarchy, set early in the response and determined by TCR avidity, can be reversed later in infection. This pattern of clonotypic progression is created by asynchronous expansion of distinct CD4^+^ T-cell clonotypes, according to antigen reactivity. Importantly, CD4^+^ T-cell clonotypic progression relies on B-cell activation and antigen presentation. Thus, not only is the B-cell response to infection helped and clonally diversified by CD4^+^ T cells, it also reciprocally helps and clonally diversifies the CD4^+^ T-cell response.

## Results

### Diversity of virus-specific CD4^+^ T cells increases over time

To study the clonotypic composition of an antiviral CD4^+^ T-cell response, we used infection of wild-type (WT) B6 mice with FV. To unequivocally identify a cohort of virus-specific CD4^+^ T cells over the course of infection, we used an adoptive transfer system^22^. Mice received allotypically marked EF4.1 T CRβ-transgenic CD4^+^ T cells (∼10,000 virus-specific cells engrafted per mouse), at the time of infection. The use of endogenous TCRα chains in EF4.1 T cells generate a semi-polyclonal TCR repertoire enriched in clonotypes reactive with the F-MLV env_122–141_ epitope^21^. Importantly, pairing of the transgenic TCRβ chain with TCR Vα2 chains (encoded by *Trav14* gene segments) or Vα3 chains (encoded by *Trav9* gene segments) creates clonotypes with higher or lower functional avidity, respectively^21^,^23^.

**Figure 1:**
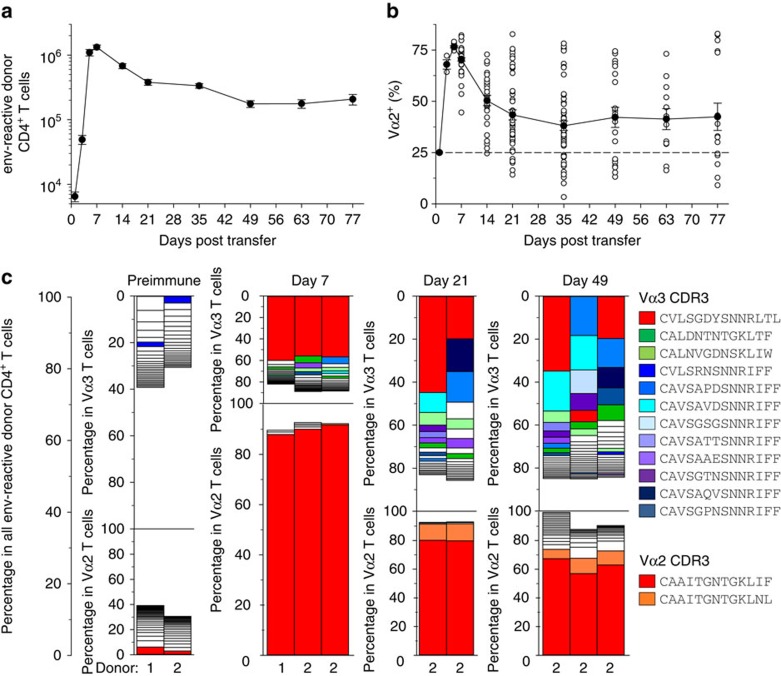
Clonotypic diversity of virus-specific CD4^+^ T cells increases over the course of FV infection. (**a**) Absolute numbers and (**b**) Vα composition of env-reactive donor EF4.1 CD4^+^ T cells in the spleens of recipient mice after adoptive T-cell transfer and FV infection (*n*=9–61 mice per time point; *P*<0.001 between Vα2 frequency on day 7 and any later time point, Mann–Whitney rank sum test). (**c**) Frequency of various Vα2 and Vα3 clonotypes in total EF4.1 CD4^+^ T cells from two uninfected donor mice (preimmune; donors 1 and 2) or in env-reactive EF4.1 CD4^+^ T cells from the spleens of recipient mice after adoptive T-cell transfer from the indicated donor and FV infection. Each bar graph represents an individual mouse. Clonotypes with frequencies <0.5% are not plotted for simplicity. Identical clonotypes within Vα2 and Vα3 subsets are marked with the same color, with red color indicating the dominant Vα2 and Vα3 clonotypes. The scale of the Vα2 and Vα3 plots are adjusted to the relative ratio of the two subsets at a given time point.

Numbers of virus-specific donor CD4^+^ T cells exhibited typical expansion and contraction kinetics (Fig. 1a). As previously observed^8^, the peak response was homogeneously dominated by high-avidity clonotypes, with Vα2 clonotypes rising to >75% on day 7 of infection (Fig. 1b). Notably, however, during progression of the response, the frequency of Vα2 clonotypes declined, on average, but varied wildly (5–95%) between identically treated individual hosts (Fig. 1b).

Although, at the population level, Vα2 clonotypes display higher functional avidity than Vα3 clonotypes, there exists the potential for considerable diversity in the semi-polyclonal repertoire of EF4.1 T cells^21^,^23^. To examine whether the observed changes in clonotypic composition were indeed caused by differences in TCR avidity between clonotypes, we measured the frequency of unique TCR clonotypes, by deep-sequencing the endogenous *Tcra* chains in virus-specific EF4.1 CD4^+^ T cells. Using *Tcra*^+/−^ EF4.1 CD4^+^ T cells, we first confirmed that replacement of Vα2 clonotypes during FV infection was not owing to potential expression of a second TCRα chain (Supplementary Fig. 1). Grouping clones into clonotypes sharing identical amino acid sequences, revealed that the peak (day 7) virus-specific Vα2 response was overwhelmingly (∼90%) dominated by a single clonotype (indicated by red color; CDR3 sequence CAAITGNTGKLIF) in all mice (Fig. 1c). This clonotype was the single largest in the entire virus-specific and non-specific Vα2 preimmune repertoire (Fig. 1c). The frequency of this dominant clonotype remained high (>60%) within virus-specific Vα2 cells throughout infection, although it was slightly reduced in comparison with its peak, and additional clonotypes were also visible at later time points (Fig. 1c). In contrast, Vα3 cells were considerably more polyclonal (Fig. 1c). The peak of the virus-specific Vα3 response in all mice examined was also dominated by a single clonotype (indicated in red color; CDR3 sequence CVLSGDYSNNRLTL), which however, comprised only ∼55% of Vα3 cells on day 7 and significantly less (between 7 and 46% in individual mice) at later time points (Fig. 1c). This particular clonotype was rare (<0.5%) in the preimmune repertoire (Fig. 1c). Notably, the virus-specific Vα3 response was also characterized by two other groups of clonotypes that exhibited distinct kinetics. The first group comprised of only two ‘public' clonotypes (indicated by green colours; CDR3 sequences CALDNTNTGKLTF and CALNVGDNSKLIW), which together made ∼10% of all virus-specific Vα3 cells, a frequency that remained remarkably stable throughout the course of infection (Fig. 1c). The second group comprised of several (>12) distinct clonotypes (indicated by blue-purple colours), all sharing the SNNRIFF motif in their CDR3 sequences, created by the use of the *Traj31* segment (Fig. 1c). The latter group contained clonotypes that were not always shared between separate mice, but collectively increased in frequency to 30–50% of the virus-specific Vα3 cells by day 49 of infection (Fig. 1c). Thus, the clonotypic diversity of virus-specific EF4.1 CD4^+^ T cells increased over time owing to the decrease in the proportion of initially dominant Vα2 clonotypes in the overall response, and increase in the representation of *Traj31*-using clonotypes within the Vα3 response.

### Clonotypic behaviour correlates with self-reactivity

**Figure 2:**
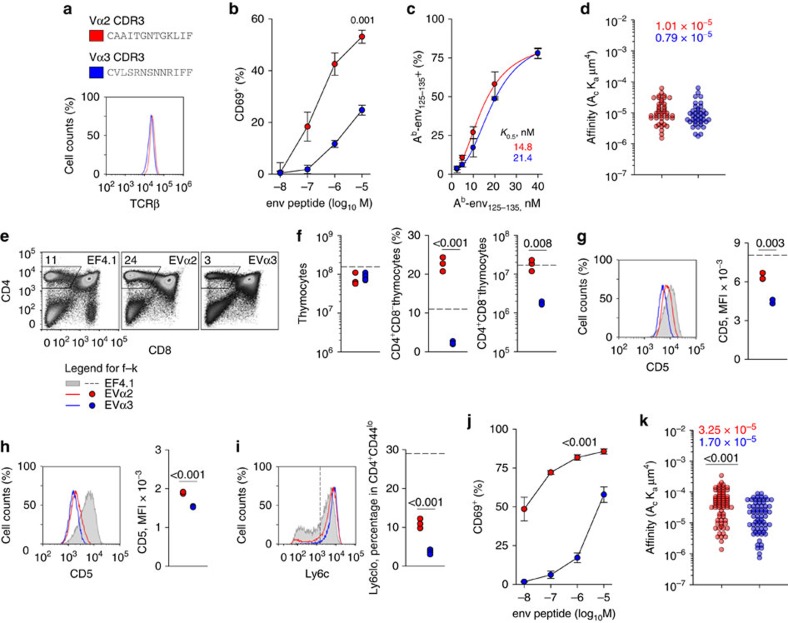
Development and reactivity with self and foreign antigen of monoclonal EVα2 and EVα3 T cells. (**a**) CDR3 sequences and TCRβ expression levels in two hybridoma cells lines representing the indicated clonotypes. (**b**) Responsiveness of the same hybridoma cells lines as in **a** to overnight stimulation with env_122–141_ peptide, measured by CD69 upregulation (*n*=2). (**c**) Frequency of A^b^-env_125–135_ tetramer-positive cells, at different tetramer concentration, following staining of the same hybridoma cells lines as in **a** and normalized for TCR expression. Symbols represent the means (±s.e.m., *n*=2) of experimental data, whereas lines are fits using a four-parameter Hill equation. Numbers next to the regression lines denote apparent affinity (*K*_0.5_) estimates. (**d**) Effective two-dimensional TCR affinities for A^b^-env_125–135_ of T-cell hybridomas measured by the micropipette adhesion frequency assay and normalized by TCR surface density. Each individual data point represents the affinity of a single T cell. Numbers in the plot represent the effective affinity geometric mean of the population. (**e**) Flow cytometric analysis of thymocyte development in EF4.1, EVα2 and EVα3 TCR-transgenic mice. (**f**) Absolute number of total thymocytes and frequency and number of CD4 single-positive thymocytes in the same mice as in **e**. (**g**) CD5 levels in post-selection (CD4^+^CD8^−^TCR^high^) thymocytes from the same mice as in **e**. (**h**) CD5 levels in naive splenic CD4^+^ T cells from EVα2 and EVα3 mice. (**i**) Ly6c levels in naive splenic CD4^+^ T cells from the same mice. In **f**–**i**, each symbol represents an individual mouse from one representative of two experiments. Horizontal dashed lines represent the mean values for control EF4.1 mice. (**j**) Responsiveness of EVα2 and EVα3 T cells to overnight env_124–138_ peptide stimulation. Responses were measured by CD69 upregulation and are plotted as means (±s.e.m., *n*=3–4). (**k**) Effective two-dimensional TCR affinities for A^b^-env_125–135_ of primary T cells measured by the micropipette adhesion frequency assay and normalized by TCR surface density, and plotted as described in **d**.

Our previous studies indicated lower functional avidity for viral antigen in Vα3 than in Vα2 clonotypes^21^,^23^, but were limited to the bulk Vα2 and Vα3 populations. To further investigate differences between unique clonotypes using more accurate measures of TCR affinity, we used hybridoma cell lines representing the two major types of behaviour during the response, ‘red' Vα2 and *Traj31*-using ‘blue-purple' Vα3 (Fig. 2a). These clones were comparably abundant in the preimmune repertoire of EF4.1 mice (Fig. 1c, preimmune) and exemplified the behaviour of other similar clones (Fig. 1c).

The two clones expressed comparable TCR levels (Fig. 2a) and their stimulation with env peptide *in vitro* reproduced the difference in functional avidity between the bulk Vα2 and Vα3 populations (Fig. 2b). Surprisingly, however, the functional avidity of these two clones correlated weakly with binding affinity of the TCR to env pMHC complexes (Fig. 2c). Moreover, precise measurement of two-dimensional TCR affinity for A^b^-env_125–135_ using a micropipette adhesion frequency assay^24^,^25^, revealed only minor differences in effective TCR affinities between the two clones (Fig. 2d).

In addition to TCR affinity for antigen, self-reactivity has recently been shown to strongly influence the sensitivity of CD4^+^ T cells to antigenic stimulation^11^,^12^. Therefore, we examined if differences in functional avidity between the selected Vα2 and Vα3 clones reflected differences in self-reactivity. The genes encoding the TCRα chains of these two clones were used to generate two TCRαβ doubly transgenic mouse strains (referred to as EVα2 and EVα3, respectively) also expressing the same TCRβ as the TCRβ-transgenic EF4.1 mice. T-cell development proceeded normally in both EVα2 and EVα3 mice, but was considerably more efficient in the former (Fig. 2e,f). Indeed, although total thymocyte numbers were near-normal in both monoclonal TCR-transgenic strains, the frequency and absolute number CD4 single-positive thymocytes was significantly higher in EVα2 mice (Fig. 2e,f), indicating more efficient positive selection. Consistent with this notion, levels of CD5, which correlate with the strength of self-reactivity, were significantly higher in EVα2 than in EVα3 post-selection (CD4^+^CD8^−^TCR^high^) thymocytes (Fig. 2g). In comparison with cells from the parental EF4.1 strain, which expressed normal CD5 levels (Supplementary Fig. 2a), EVα2 and EVα3 thymocytes expressed 63 and 34%, respectively, of normal CD5 levels. Peripheral CD4^+^ T-cell numbers, which also reflect the degree of self-reactivity^26^, were comparable to the WT levels in EVα2 mice, but significantly reduced in EVα3 mice (Supplementary Fig. 2b). Mature CD4^+^ T cells were homogenously naive (CD44^low^CD25^−^) in both monoclonal TCR-transgenic strains and expressed comparable TCR levels (Supplementary Fig. 2c). CD5 levels were reduced in both EVα2 and EVα3 peripheral T cell in comparison with thymocytes and with WT T cells (Fig. 2h). This was likely because of the relative paucity of MHC II-expressing cells in the periphery of these B-cell-deficient *Rag1*^−/−^ EVα2 and EVα3 mice. Indeed, comparison of EVα2 T cells from B-cell-deficient *Rag1*^−/−^ or B-cell-sufficient *Rag1*^+/+^ mice revealed a specific role for B cells in maintaining CD5 levels in peripheral, but not thymic CD4^+^ T cells (Supplementary Fig. 2d,e). Nevertheless, CD5 levels remained significantly higher in EVα2 than in EVα3 peripheral T cells (Fig. 2h) and loss of Ly6C expression, which signifies higher self-reactivity^27^, marked a higher proportion of EVα2 than in EVα3 peripheral T cells (Fig. 2i). Phenotypic differences between monoclonal EVα2 and EVα3 T cells correlated remarkably well with functional avidity, which was reflected in their sensitivity to *in vitro* env peptide stimulation (Fig. 2j). In contrast, two-dimensional TCR affinity for A^b^-env_125–135_ differed only by a factor of two between the two clones (Fig. 2k). Although this difference was statistically significant, it was too small to account for the disparity in antigen reactivity. Thus, functional avidity of EVα2 and EVα3 T cells better correlated with self-reactivity than with affinity for antigen.

### T-cell clonal composition depends on infection kinetics

**Figure 3:**
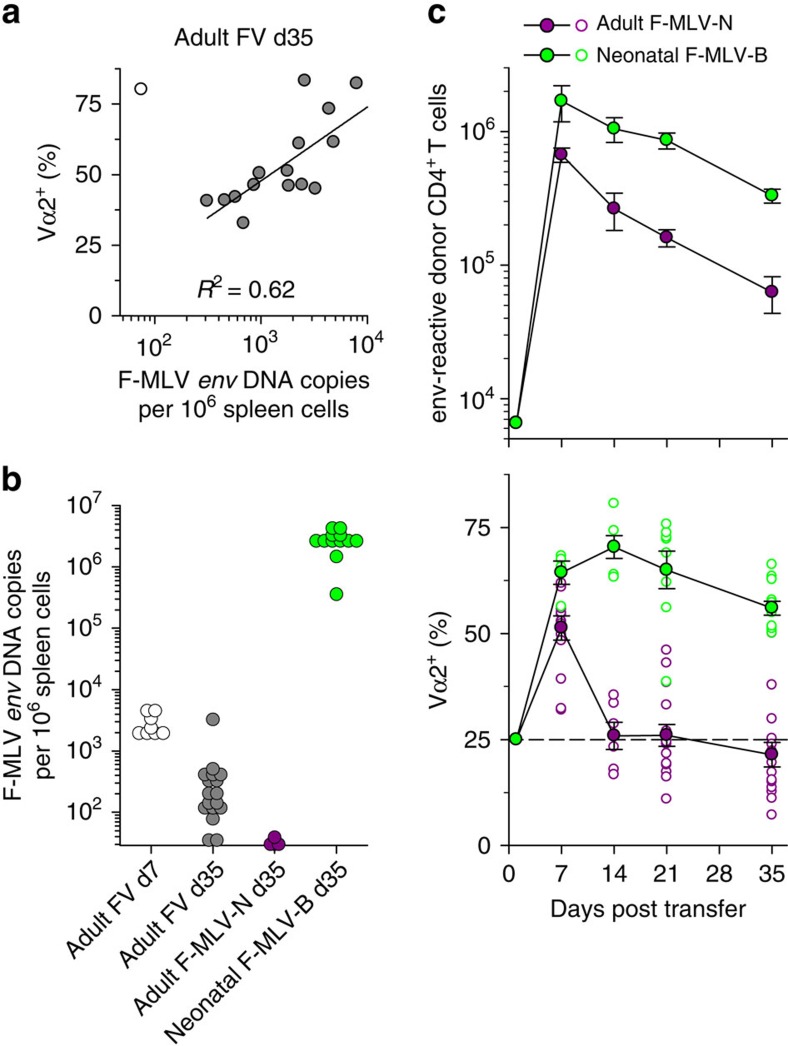
Effect of infection kinetics on the clonal composition of virus-specific CD4^+^ T cells. (**a**) Frequency of Vα2^+^ cells within env-reactive donor EF4. 1 CD4^+^ T cells plotted against copies of F-MLV *env* DNA in the spleens of recipient mice 35 days after adoptive T-cell transfer and FV infection. Symbols represent individual mice. The mouse represented with an open symbol was excluded from the regression analysis. (**b**) Copies of F-MLV *env* DNA in the spleens of recipient mice that were infected as adults with FV or F-MLV-N or as neonates with F-MLV-B. All recipients received EF4.1 CD4^+^ T cells as adults and analysed at the indicated time point after T-cell transfer. Each symbol is an individual mouse. The dashed line denotes the detection limit. (**c**) Absolute numbers (top) and Vα composition (bottom) of env-reactive donor EF4.1 CD4^+^ T cells in the spleens of recipient mice after T-cell adoptive transfer in adult mice either infected with F-MLV-N at the time of T-cell transfer or with F-MLV-B as neonates (*n*=4–15 mice per time point; *P*<0.001 between the two types of host on days 14–35, Mann–Whitney rank sum test). Closed symbols are the means (±s.e.m.); open symbols are individual mice; the dashed line represents the frequency of Vα2^+^ cells in preimmune env-reactive EF4.1 CD4^+^ T cells.

In B6 mice, acute FV infection is followed by chronic low-level infection, which can be ultimately cleared with kinetics that likely differ most significantly between FV-infected hosts. An effect of viral load on clonotypic composition was suggested by a strong correlation between F-MLV DNA copy numbers in the spleens of FV-infected recipients and frequency of Vα2 clonotypes in virus-specific CD4^+^ T cells 35 days post infection (Fig. 3a). However, this positive correlation (Fig. 3a) argued against relative loss of Vα2 clonotypes owing to excessive activation, as suggested by studies in another TCR-transgenic system^10^,^11^. To examine whether viral load directly determined the clonotypic composition of the antiviral T-cell response, we infected adult B6 mice with N-tropic F-MLV (F-MLV-N), which is restricted by the product of the *Fv1*^b^ allele and quickly eliminated from these mice^28^ (Fig. 3b). In addition, we infected neonatal B6 mice with B-tropic F-MLV (F-MLV-B), which results in life-long high-level infection (Fig. 3b) and used them 6–8 weeks later as T-cell hosts. The magnitude of the EF4.1 CD4^+^ T-cell response to adult F-MLV-N or neonatal F-MLV-B infection was proportional to the degree of viral replication in these two infections (Fig. 3c). However, the frequency of Vα2 clonotypes in virus-specific donor CD4^+^ T cells declined quickly and homogeneously in all recipients transiently infected with F-MLV-N, whereas it remained very high in all recipients with non-resolving F-MLV-B infection (Fig. 3c). These results demonstrated that the clonotypic evolution of the EF4.1 CD4^+^ T-cell response to retroviral infection was directly determined by the kinetics of viral replication and that higher antigen levels promoted, rather than hindered, clonotypes with higher antigen sensitivity.

### B cells mediate clonal replacement in CD4^+^ T cells

**Figure 4:**
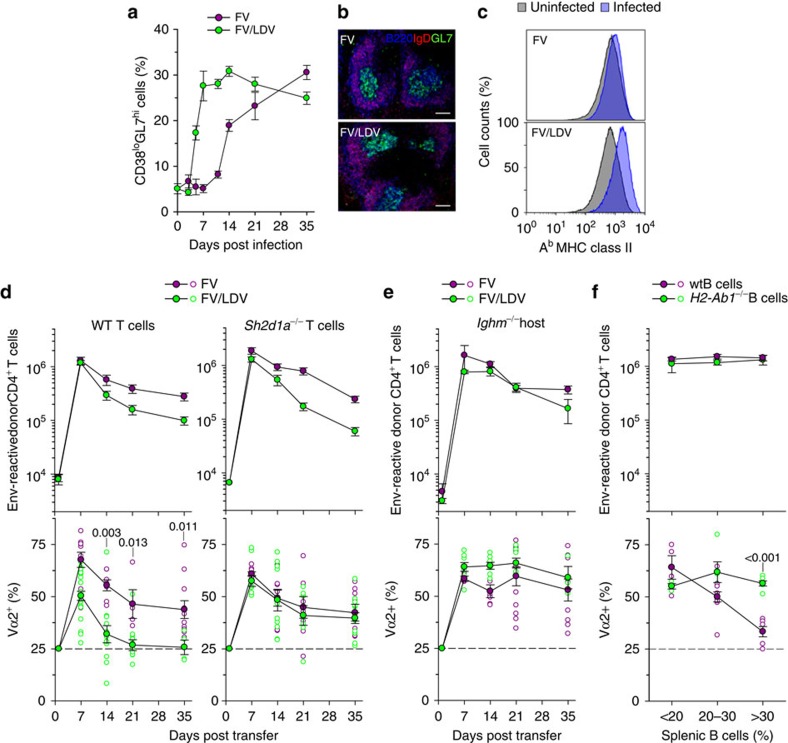
Clonal replacement in virus-specific CD4^+^ T cells requires cognate B-cell interaction. (**a**) Frequency of germinal center-phenotype (CD38^lo^GL7^hi^) cells in CD19^+^IgD^lo^ splenic B cells over the course of FV infection or FV/LDV coinfection (*n*=4–13; *P*<0.002 between the two infections on days 5–14, Mann–Whitney rank sum test). (**b**) Germinal center (B220^+^IgD^−^GL7^+^) presence in the spleen of the same mice in **a** 35 days post infection. The scale bar is 250 μm. (**c**) MHC II expression in B220^+^CD19^+^ B cells from the spleen of the same mice in **a** 7 days post infection, compared with uninfected mice. (*P*=0.0016 between the two infections, Student's *t*-test). (**d**) Absolute numbers (top) and Vα composition (bottom) of WT (left) or *Sh2d1a*^−/−^ (right) env-reactive donor EF4.1 CD4^+^ T cells in the spleens of recipient mice after adoptive T-cell transfer and FV infection (*n*=3–8 mice per time point) or FV/LDV coinfection (*n*=3–9 mice per time point). (**e**) Absolute numbers (top) and Vα composition (bottom) of env-reactive donor EF4.1 CD4^+^ T cells in the spleens of WT or *Ighm*^−/−^ recipient mice after adoptive T-cell transfer and FV infection (*n*=4–11 mice per time point) or FV/LDV coinfection (*n*=4–23 mice per time point). (**f**) Absolute numbers (top) and Vα composition (bottom) of env-reactive donor EF4.1 CD4^+^ T cells in the spleens of *Ighm*^−/−^ mice previously reconstituted with WT (*n*=18) or *H2-Ab1*^−/−^ B cells (*n*=14) 14 days after adoptive T-cell transfer FV/LDV coinfection, plotted against the level of B-cell reconstitution. In **d**–**f**, closed symbols are the means (±s.e.m.); open symbols are individual mice.

T cells recognize viral antigens only through APCs. Thus, we reasoned that the effect of viral antigen availability on the composition virus-specific CD4^+^ T cells involved APCs. In addition to acting as APCs to T cells, B cells mount their own antigen-specific and non-specific response to viral infection with distinct temporal kinetics. In response to FV infection, the frequency of germinal center CD38^lo^GL7^hi^ cells within IgD^lo^CD19^+^ B cells slowly increased starting from day 10 post infection and continuing until the last time point studied (day 35; Fig. 4a,b). Interestingly, the rise in activated B cells temporally matched the decline in Vα2 clonotypes in virus-specific donor CD4^+^ T cells in FV infection (Fig. 1b and Fig. 4a). To examine a possible link, we compared hosts infected with FV with those coinfected with FV and lactate dehydrogenase-elevating virus (LDV), which has been previously shown to induce rapid polyclonal B-cell activation and enhance FV replication^29^. As expected, FV-LDV coinfection greatly accelerated the rise in germinal center B cells and induced higher expression of MHC II on all B cells (Fig. 4a,c). Importantly, FV-LDV coinfection resulted in significantly faster decline in the frequency of Vα2 clonotypes in virus-specific EF4.1 CD4^+^ T cells, than FV infection (Fig. 4d), supporting a role for polyclonally activated B cells.

As coinfection with LDV is likely to affect multiple processes during the response to FV, we next examined if the accelerated Vα2 clonotype decline required T cell–B cell interaction. To this end, we used EF4.1 CD4^+^ T cells deficient in *Sh2d1a* (encoding SAP, multiple signalling lymphocyte activation molecule-associated protein), which is required for prolonged CD4^+^ T cell–B cell contacts^30^. Although SAP deficiency in donor CD4^+^ T cells had little effect on their numerical response to FV infection or FV-LDV coinfection, it completely negated the accelerated decline of Vα2 clonotypes in virus-specific EF4.1 CD4^+^ T cells by LDV (Fig. 4d).

As T-cell-specific SAP deficiency shortens, but does not abolish T cell–B cell interaction^30^, we next used mice deficient in B cells (*Ighm*^−/−^) as hosts. In the complete absence of B cells, high-avidity Vα2 clonotypes were induced and maintained at high levels throughout FV infection or FV-LDV coinfection (Fig. 4e). To dissociate a possible effect of B cells as APCs from other B-cell-dependent processes in the host (for example, splenic structure) we reconstituted non-irradiated *Ighm*^−/−^ hosts with either MHC II-sufficient or -deficient B cells (Supplementary Fig. 3). Following FV-LDV coinfection, MHC II-sufficient, but not -deficient B cells significantly reduced the frequency of high-avidity Vα2 clonotypes, in a dose-dependent manner, without affecting the total virus-specific donor CD4^+^ T-cell numbers (Fig. 4f). Therefore, clonotypic replacement of Vα2 virus-specific CD4^+^ T cells over the course of infection required cognate T cell–B cell interaction.

### CD4^+^ T-cell clonal replacement affects all Th subsets

**Figure 5:**
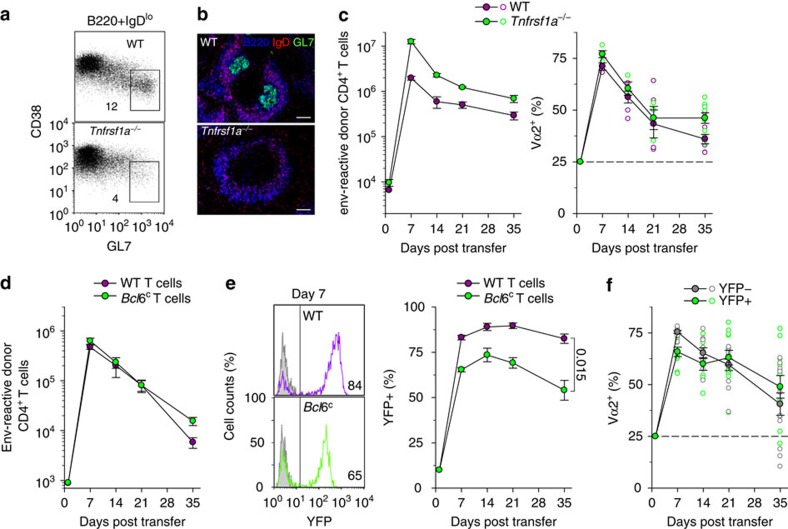
Clonal replacement in virus-specific CD4^+^ T cells independently of Bcl6-dependent Tfh differentiation or germinal center reaction. (**a**) Frequency of germinal center-phenotype (CD38^lo^GL7^hi^) cells in CD19^+^IgD^lo^ splenic B cells 35 days after FV infection of WT or *Tnfrsf1a*^−/−^ mice (*n*=3). (**b**) Germinal center (B220^+^IgD^−^GL7^+^) presence in the spleen of the same mice in **a**. The scale bar is 250 μm. (**c**) Absolute numbers (left) and Vα composition (right) of env-reactive donor EF4.1 CD4^+^ T cells in the spleens of WT or *Tnfrsf1a*^−/−^ recipient mice after adoptive T-cell transfer and FV infection (*n*=3–9 mice per time point). (**d**) Absolute numbers of WT or Bcl6-conditional (*Bcl6*^c^) env-reactive donor EF4.1 CD4^+^ T cells, additionally carrying the *Tnfrsf4*^Cre^ and *Gt(ROSA)26Sor*^YFP^ alleles, in the spleens of recipient mice after adoptive T-cell transfer and FV infection (*n*=3–9 mice per time point). (**e**) YFP expression on day 7 (left) and frequency of YFP^+^ cells over time (right) in the same env-reactive donor EF4.1 CD4^+^ T cells as in **d**. (**f**) Vα2 frequency in YFP^+^ and YFP^−^ Bcl6-conditional env-reactive donor EF4.1 CD4^+^ T cells as in **d**. In **c**–**f**, closed symbols are the means (±s.e.m.); open symbols are individual mice.

Follicular helper (Tfh) cells rely on B-cell interaction and the germinal center response for their differentiation and persistence. Thus, it was theoretically possible that B cells promoted Tfh differentiation of particular T-cell clonotypes selectively, whose representation was subsequently proportional to the magnitude of the germinal center response. To test the requirement for the physical germinal center structures, we used *Tnfrsf1a*-deficient hosts, which show incomplete segregation of T-cell and B-cell areas in secondary lymphoid organs and are unable to mount a germinal center response (Fig. 5a,b). In comparison with those in WT hosts, higher numbers of virus-specific donor CD4^+^ T cells were recovered from *Tnfrsf1a*-deficient hosts throughout the response (Fig. 5c). Importantly, however, the Vα2 and Vα3 composition of donor CD4^+^ T cells was comparable between the two types of hosts (Fig. 5c), ruling out a requirement for germinal centres in the clonotypic evolution of virus-specific CD4^+^ T cells.

To directly examine if replacement of Vα2 clonotypes was owing to preferential differentiation into Tfh cells, we conditionally ablated *Bcl6* in donor CD4^+^ T cells, precluding their Tfh differentiation. EF4.1 CD4^+^ T cells were isolated from mice carrying a Cre-conditional *Bcl6* (*Bcl6*^c^) and a YFP reporter (*Gt(ROSA)26Sor*^YFP^) allele and also expressed Cre under the control of the *Tnfrsf4* promoter (*Tnfrsf4*^Cre^), thus initiating YFP expression and *Bcl6* deletion only upon T-cell activation^31^ (Fig. 5d,e). EF4.1 CD4^+^ T cells with either WT or *Bcl6*^c^ alleles, exhibited typical response kinetics upon transfer into FV-infected hosts (Fig. 5d). However, the frequency of YFP^+^ cells in virus-specific donor CD4^+^ T cells was significantly lower in EF4.1 CD4^+^ T cells with the *Bcl6*^c^ than the WT allele (Fig. 5e), indicating that Bcl6 is required for the persistence of at least some virus-specific CD4^+^ T cells. Nevertheless, the clonotypic composition of YFP^+^ (*Bcl6*-deleted) virus-specific donor CD4^+^ T cells was comparable with that of the YFP^−^ fraction (Fig. 5f). Thus, preventing Bcl6-dependent Tfh differentiation did not prevent the switch from Vα2 to Vα3 clonotypes (*P*=0.016 Vα2 frequency in YFP^+^ cells between days 7 and 35, Mann–Whitney rank sum test).

These findings argued against the possibility that clonotypic replacement of Vα2 clonotypes with *Traj31*-using Vα3 clonotypes over the course of FV infection was owing to selective differentiation into the Tfh subset, whose long-term stability may differ from that of other Th subsets. This led us to consider an alternative hypothesis: instead of all virus-specific clonotypes engaging synchronously and *Traj31*-using Vα3 clonotypes surviving longer than other clonotypes, it was possible that *Traj31*-using Vα3 clonotypes were simply recruited with delayed kinetics.

### Asynchronous expansion of high- and low-avidity clonotypes

**Figure 6:**
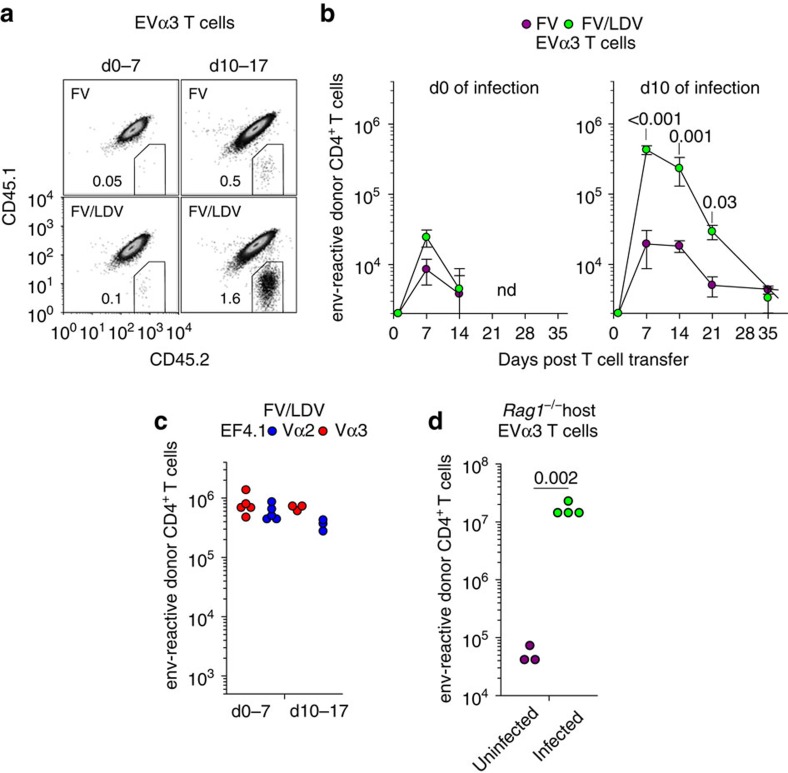
Asynchronous expansion of higher- and lower-avidity clonotypes. (**a**) Flow cytometric detection of CD45.2^+^ donor EVα3 *Rag1*^−/−^ *Emv2*^−/−^ CD4^+^ T cells and CD45.1^+^CD45.2^+^ host CD4^+^ T cells following transfer either on the day of infection (d0 of infection, d0–7) or 10 days after infection (d10 of infection, d10–17) with FV or FV/LDV. In both setups, T-cell expansion was analysed 7 days after transfer (*n*=5–9). (**b**) Absolute number of env-reactive donor EVα3 CD4^+^ T cells recovered from the spleens of the same recipients described in **a** over the course of FV infection or FV/LDV coinfection (nd, not detected). (**c**) Absolute number of Vα2 or Vα3 env-reactive donor EF4.1 CD4^+^ T cells recovered from the spleens of FV/LDV infected recipients 7 days after transfer either on the day of infection (d0–7) or 10 days after infection (d10–17). (**d**) Numbers of EVα3 T cells recovered 7 days after transfer from the spleens of lymphocyte-deficient *Rag1*^−/−^ *Emv2*^−/−^ recipients that were either left uninfected or were F-MLV-B-infected. Each symbol represents an individual mouse.

The extensive variability in the frequency of Vα3 clonotypes between separate hosts during FV infection (Fig. 1b), and the ‘private' nature of distinct *Traj31*-using Vα3 clonotypes expanding in different hosts (Fig. 1c) hindered kinetic analyses of the deep-sequencing data. To establish conclusively the kinetics of recruitment and expansion of a *Traj31*-using Vα3 clonotype in response to FV infection, we transferred cohorts of allotypically marked monoclonal EVα3 T cells into WT hosts that were infected with FV or coinfected with FV and LDV, either 10 days previously or on the day of T-cell transfer (Fig. 6a,b). EVα3 T cells transferred at the time of FV infection or FV/LDV coinfection expanded minimally 7 days later and became undetectable by day 21 post transfer (Fig. 6a,b). Also, EVα3 T cells transferred on day 10 of FV infection expanded modestly 7 days after transfer and contracted to low numbers thereafter (Fig. 6a,b). This pattern contrasted with strong expansion (>100-fold) of EVα3 T cells 7 days after transfer into hosts coinfected with FV and LDV 10 days previously (Fig. 6a,b), when B-cell activation had peaked (Fig. 4a). For comparison, EF4.1T cells were also transferred into hosts that were coinfected with FV and LDV, either 10 days previously or on the day of T-cell transfer, and maximal expansion of both Vα2 and Vα3 clonotypes was already observed the first 7 days of infection (Fig. 6c). These results demonstrated that EVα3 T cells were recruited later than higher-avidity virus-specific clonotypes and only in conditions where B cells were activated.

Later recruitment, as opposed to longer survival, of EVα3 T cells further argued that B-cell presentation did not necessarily instill qualitative differences in CD4^+^ T-cell clonotypes, but rather expanded the number of available APCs to the point where clonotypic competition was alleviated and lower-avidity T-cell clonotypes could be expanded. Indeed, recruitment and expansion of lower-avidity T-cell clonotypes depended on the availability of activated B cells only in the presence of higher-avidity T-cell competitors. In contrast to their lack of expansion in FV-infected WT host (Fig. 6a,b), EVα3 T cells expanded markedly when transferred into FV-infected *Rag1*^−/−^ hosts (Fig. 6d). These results were consistent with a quantitative, rather than qualitative effect of B-cell presentation on the expansion of lower-avidity CD4^+^ T-cell clonotypes, whereby the B-cell response gradually increases APC availability, which progressively recruits lower-avidity T-cell clonotypes, thus diversifying the CD4^+^ T-cell response.

### B cells promote TCR diversity in various T-cell responses

**Figure 7:**
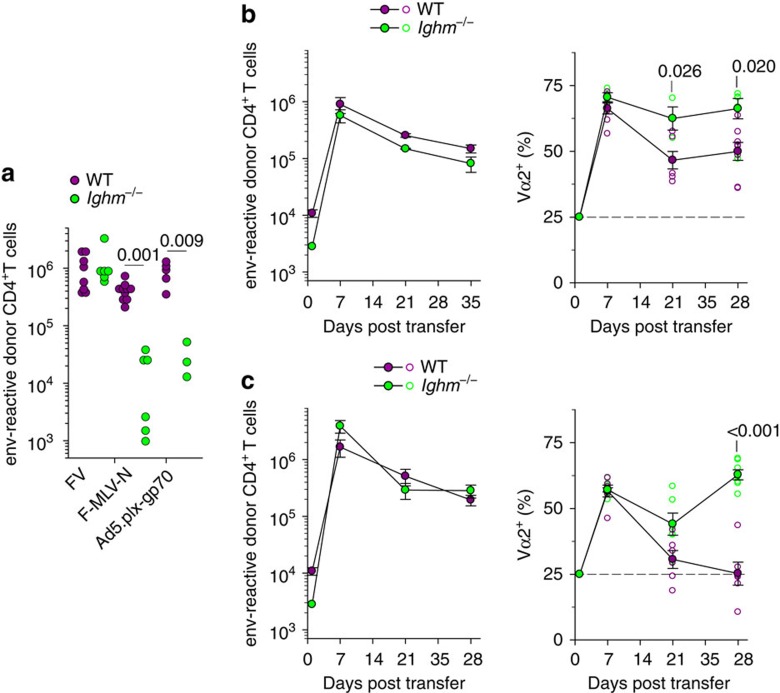
B-cell-dependent expansion of lower-avidity CD4^+^ T-cell clonotypes in diverse infection or immunization settings. (**a**) Absolute number of env-reactive donor EF4.1 CD4^+^ T cells 7 days after transfer into WT or *Ighm*^−/−^ recipient mice infected either with FV or F-MLV-N or immunized with Ad5.pIX-gp70. Each symbol is an individual mouse. (**b**) Absolute numbers (left) and Vα composition (right) of env-reactive donor EF4.1 CD4^+^ T cells in the spleens of WT or *Ighm*^−/−^ recipient mice after adoptive T-cell transfer and FBL-3 tumor challenge (*n*=3–8 mice per time point). (**c**) Absolute numbers (left) and Vα composition (right) of env-reactive donor EF4.1 CD4^+^ T cells in the spleens of WT or *Ighm*^−/−^ recipient mice after adoptive T-cell transfer and env_122–141_ peptide immunization (*n*=6–9 mice per time point).

Our model would also predict that following infections or immunizations where B cells are dominant APCs, lower-avidity clonotypes would be induced, in addition to higher-avidity clonotypes. To test this prediction, we compared the dependence on B cells of EF4.1 CD4^+^ T-cell priming by the replication-attenuated F-MLV-N vaccine virus and fully competent FV, which induce lower- and higher-avidity responses, respectively (Fig. 1b and Fig. 3c). Indeed, CD4^+^ T-cell priming by F-MLV-N, but not FV, was significantly compromised in the absence of B cells (Fig. 7a). Similarly, priming with a replication-defective human Adenovirus 5 (Ad5)-based vector, expressing F-MLV gp70 (Ad5.pIX-gp70), which has previously found to induce primarily lower-avidity responses^16^, was also compromised in the absence of B cells (Fig. 7a). Therefore, vaccines that rely on B-cell presentation induce lower-avidity CD4^+^ T-cell responses.

The applicability of this model was also tested in two additional immunization regimens. WT or *Ighm*^−/−^ hosts were challenged with FBL-3 cells, a FV-induced tumour cell line that expresses F-MLV gp70, but does not produce infectious viral particles^32^. Adoptively transferred EF4.1 CD4^+^ T cells responded with a high frequency of high-avidity Vα2 virus-specific CD4^+^ T-cell clonotypes in both types of host at peak (Fig. 7b). Importantly, this frequency remained significantly higher in *Ighm*^−/−^ than in WT hosts as the response progressed (Fig. 7b). Moreover, the clonotypic replacement of Vα2 virus-specific CD4^+^ T cells was observed following env_124–138_ peptide immunization of WT, but not *Ighm*^−/−^ hosts, highlighting the requirement for B cells in the process. Thus, following either tumour challenge or peptide immunization, B cells were required for the full clonotypic diversity of the CD4^+^ T-cell response.

### B cells balance TCR diversity during T-cell reconstitution

**Figure 8:**
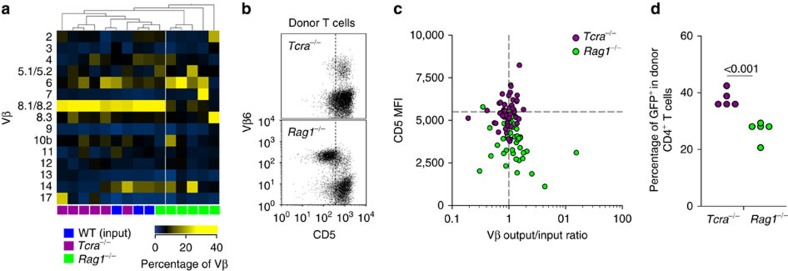
Balanced TCR diversity during CD4^+^ T-cell reconstitution requires B cells. (**a**) Heat-map of hierarchically-clustered WT donors and *Tcra*^−/−^ or *Rag1*^−/−^ *Emv2*^−/−^ recipients of purified CD4^+^ T cells according to the frequency of the indicated Vβ family before (WT (input)) or 21 days after T-cell reconstitution (*Tcra*^−/−^ or *Rag1*^−/−^). Each column is an individual mouse. (**b**) Flow cytometric detection of CD5 and Vβ6 expression in donor CD4^+^ T cells from the same recipients as in **a**. (**c**) CD5 median fluorescent intensity (MFI) plotted against the divergence from the input frequency of each detected Vβ family in donor CD4^+^ T cells from the same recipients as in **a**. Only three recipients are plotted for clarity. The horizontal dashed line represents the CD5 MFI in control cells (*P*<0.001 between CD5 levels in T cells from the two types of host, Mann–Whitney rank sum test). (**d**) Frequency of GFP^+^ cells in donor CD4^+^ T cells 21 days after T-cell reconstitution of *Tcra*^−/−^ or *Rag1*^−/−^ *Emv2*^−/−^ (*Rag1*^−/−^) recipients with purified CD4^+^ T cells from Nur77-GFP transgenic donors. Each symbol represents an individual mouse.

Our model implied that B-cell presentation should promote TCR diversity not only in response to infection or immunization, but also in any setting where CD4^+^ T-cell clonotypic competition is likely to operate based on TCR avidity. To examine if this were the case, we monitored TCR repertoire skewing during CD4^+^ T-cell reconstitution of lymphopenic hosts, which selects distinct clonotypes according to TCR avidity. Hosts lacking only T cells (*Tcra*^−/−^) or both T and B cells (*Rag1*^−/−^) were reconstituted by adoptive transfer of fully polyclonal purified CD4^+^ T cells and usage of different families of Vβ chains was compared between the input and expanded population in the respective host 21 days later (Fig. 8a). T-cell reconstitution of *Tcra*^−/−^ hosts preserved the pattern of Vβ usage in the input population, as the two were indistinguishable by unsupervised clustering (Fig. 8a). In contrast, T-cell reconstitution of *Rag1*^−/−^ hosts resulted in considerable skewing in Vβ usage in 5 out of 6 such hosts, which clustered separately from all other hosts (Fig. 8a). TCR Vβ usage skewing was not related to differences in overall T-cell reconstitution between Tcra^−/−^ and *Rag1*^−/−^ hosts, as it also characterized hosts with comparable numbers of expanded CD4^+^ T cells (Supplementary Fig. 4). TCR repertoire skewing in *Rag1*^−/−^ hosts was owing to ‘private' expansion of distinct Vβ families in individual mice (Fig. 8a), suggestive of TCR driven responses. Moreover, levels of CD5 expression were significantly reduced in CD4^+^ T cells expanded in *Rag1*^−/−^ hosts in comparison with those expanded in *Tcra*^−/−^ hosts (Fig. 8b,c), indicating insufficient MHC II-derived signals in *Rag1*^−/−^ hosts^33^,^34^. Indeed, use of the Nur77-GFP reporter for TCR signalling^35^ revealed that CD4^+^ T cells received stronger overall TCR signals in *Tcra*^−/−^ than *Rag1*^−/−^ hosts (Fig. 8d), suggesting that B cells were necessary to provide CD4^+^ T cells with optimal TCR signal, which would be critical for the expansion of lower-avidity clonotypes. Thus, balanced representation of TCR clonotypes, at least at the level of distinct Vβ families during T-cell reconstitution, required B cells.

## Discussion

Our findings highlight the prominent role for the TCR in setting the clonotypic hierarchy during the CD4^+^ T-cell response, suggested by studies in many systems^36^. They lend support to the recent recognition of the importance of self-reactivity, rather than affinity for antigen, in determining the fate of CD4^+^ and CD8^+^ T cells during the response to foreign antigens^11^,^12^,^37^. However, they further uncover the role of B-cell presentation as a powerful T-cell-extrinsic factor that can reverse TCR sensitivity-based hierarchies. Indeed, although T-cell clonal selection driven by TCR affinity for self or foreign antigens progressively narrows the responding TCR repertoire, B cells are critical in preserving diversity of the antigen-selected TCR repertoire.

The progression of the CD4^+^ T-cell response is generally expected to enrich for high-avidity clonotypes, accentuated by diminishing antigen levels, a notion that is supported by experimental data^10^,^38^,^39^,^40^. The central importance of TCR signal strength in memory formation would gradually reduce the diversity to a few or theoretically the one clonotype with the highest avidity. However, enrichment for high-avidity clonotypes is not consistent with either continuous clonal replacement during chronic T-cell responses^41^ or following secondary responses^10^,^42^,^43^. Moreover, memory CD4^+^ T-cell populations with lower overall avidity than the primary response have also been found in response to *Salmonella typhimurium* infection^44^, and a mouse adenocarcinoma-associated antigen^45^.

The contribution of low-avidity CD4^+^ T cells to the antigen-specific response may be underestimated owing to technical difficulties in detecting such clonotypes by pMHC II tetramers^23^,^24^. Nevertheless, an equally plausible explanation for variable participation of lower-avidity CD4^+^ T-cell clonotypes is likely related to the nature of antigen and the range of TCR affinities it elicits. T-cell-extrinsic effects on TCR clonotypic composition will be particularly pronounced when TCR affinity for antigen might not be strong enough to overcome these effects. For example, self-tolerance is likely to reduce the overall avidity of T-cell responses to tumour-associated or self-antigens or to pathogen antigens with significant similarity with self, such as retroviruses^1^,^2^.

Another important variable in studies of TCR repertoire changes over the course of the CD4^+^ T-cell response is the definition of the exact property of antigen-selected TCRs. Although there is general consensus that CD4^+^ T cells are selected on the basis of TCR signal strength in response to antigen, the underlying factors responsible for differences in TCR sensitivity are still a matter of debate. TCR binding kinetics to antigenic pMHC II complexes have traditionally been considered as the dominant factor^9^,^46^. However, the importance of TCR self-reactivity in setting TCR responsiveness to antigen is increasingly appreciated^11^,^12^,^37^. Strong self-reactivity has been suggested to select for TCRs with improved binding kinetics to antigenic pMHC^12^ or to maintain a more efficient state of TCR signalling capacity, irrespective of affinity to antigenic pMHC^11^. Our results with the EVα2 and EVα3 TCRs are consistent with the latter notion, whereby differences in functional avidity and biological response correlate with self-reactivity rather than affinity to antigen^11^. However, as a direct mechanistic link between self-reactivity and antigen sensitivity was not examined in the current study, our results do not prove that differences in antigen sensitivity are indeed caused by differences in self-reactivity.

Although naive CD4^+^ T-cell priming is thought to be initiated by DCs in the T-cell zone of secondary lymphoid organs, the contribution to maximal T-cell expansion of additional APCs and B cells in particular, is well-recognized^47^. Our findings uncover an additional dimension to the antigen-presenting contribution of B cells, namely the dependence on the B-cell response itself. Indeed, B-cell-mediated expansion of lower-avidity CD4^+^ T-cell clonotypes was proportional to the degree of B-cell activation. Polyclonal B-cell activation is a feature of many viral infections or autoimmune diseases^48^,^49^. Notably, progression of human immunodeficiency virus-1 infection is characterized by stepwise B-cell hyperactivation^50^, asynchronous expansion of CD8^+^ T cells specific to distinct epitopes^51^ and increase in virus-specific CD4^+^ T cells with low functional avidity^52^. The observation that germinal center formation was redundant in this process and that expansion of lower-avidity CD4^+^ T-cell clonotypes correlated simply with the overall number of activated B cells in bone marrow chimeras, argue against a requirement for a specific B-cell subset. Moreover, efficient priming of lower-avidity CD4^+^ T-cell clonotypes in the absence of B cells, when higher-avidity T-cell competitors were also absent, further argues against a unique type of antigen presentation by B cells. Instead, these findings suggest that B cells, which outnumber DCs at steady-state by two orders of magnitude^53^ and which further expand in response to polyclonal and antigen-specific stimuli during infection, provide the necessary abundance of antigenic stimulation to allow priming and expansion of all clonotypes irrespective of TCR avidity.

Our findings also indicate an essential role for B cells in maintenance of a diverse and balanced TCR repertoire during CD4^+^ T-cell reconstitution of lymphopenic hosts, typically driven by relatively low-avidity interactions with self- and environmental-antigens and selecting for clonotypes that receive the strongest TCR signal^4^. These results, thus, further support the idea that B cells facilitate the expansion of lower-avidity clonotypes in general. Similarly, B cells may be critically required to initiate CD4^+^ T-cell responses to an autoantigen, which would be typically low-avidity. Interestingly, non-obese diabetic mice do not develop autoimmunity if they are rendered deficient in B cells^54^, and B-cell reconstitution is required for the formation of a diverse TCR repertoire of CD4^+^ T-cell infiltrating the pancreas^55^. Although the overall avidity for pancreatic autoantigens of the different TCR repertoires was not examined^55^, these studies offer further support to the notion that B cells increase the diversity of responding CD4^+^ T cells.

B cells have long been suspected as drivers of the diversification of the CD4^+^ T-cell response^56^ and this study provides clear evidence of their involvement. In contrast to the intrinsic TCR affinity of distinct clonotypes and the clonotypic diversity in an individual, manipulating the degree of B-cell activation or antigen presentation to CD4^+^ T cells, might provide a more amenable way of controlling the antigen-specific TCR repertoire during infection, vaccination or autoimmunity.

## Methods

### Mice

Inbred B6 and CD45.1^+^ congenic B6 (B6.SJL-*Ptprc*^*a*^ *Pep3*^*b*^/BoyJ) mice were originally obtained from The Jackson Laboratory (Bar Harbor, ME, USA). TCRβ-transgenic EF4.1 mice^21^, Rag1-deficient (*Rag1*^−/−^) mice^57^, B-cell-deficient (*Ighm*^−/−^) mice^58^, SAP-deficient (*Sh2d1a*^−/−^) mice^59^, TCR α-deficient (*Tcra*^−*/*−^) mice^60^, MHC II-deficient (*H2-Ab1*^−/−^) mice^61^, TNF receptor I-deficient (*Tnfrsf1a*^−/−^) mice^62^, mice with an activatable YFP gene targeted into *Gt(ROSA)26Sor* (*R26*) locus^63^, mice with a targeted insertion of Cre recombinase into the *Tnfrsf4* locus^64^ (*Tnfrsf4*^*Cre*^), mice with a conditional *Bcl6* allele^65^ (*Bcl6*^c^), endogenous ecotropic MLV-deficient (*Emv2*^−/−^) mice^23^ and Nur77-GFP transgenic mice^35^ were all on the B6 genetic background. TCRαβ-transgenic EVα2 and EVα3 mice were created by conventional transgenesis (Supplementary Fig. 5). Briefly, cDNAs encoding the TCRα and TCRβ chains of the H5 and H18 env-specific CD4^+^ T-cell clones, respectively, from the polyclonal repertoire of EF4.1 mice^23^, were cloned and inserted into the hCD2-VA expression cassette^21^. Each of these constructs was mixed with a construct encoding the TCRβ chain of EF4.1 mice and integrated into the DNA of fertilized B6 oocytes following pronuclear microinjection. Transgenic founders were identified by flow cytometry and genotyping for the presence of the TCRα and TCRβ transgenes. These new transgenic mice were crossed to *Rag1*^−/−^ mice, to preclude rearrangement and subsequent expression of productive endogenous TCR genes. They were additionally rendered deficient in *Emv2*, a single germ-line integration of a MLV found in B6 mice, and free from any *Emv2*-derived infectious MLVs that spontaneously arise in immunodeficient mice and have the potential to affect MLV-specific T-cell development and subsequent response^66^. Male or female mice were used in separate experiments and were gender-matched within experiments. Mice were used at 8–12 weeks of age, with the exception of neonatal infection, which was carried out on 1–2 day-old mice. All animal experiments were approved by the ethical committee of the Francis Crick institute, and conducted according to local guidelines and UK Home Office regulations under the Animals Scientific Procedures Act 1986 (ASPA).

### Retroviral infection and immunization

The FV used in this study was a retroviral complex of a replication-competent B-tropic F-MLV and a replication-defective SFFV. Stocks were propagated *in vivo* and prepared from the spleen of infected mice. A pool of 20 LDV-free BALB/c mice was infected with FV, spleens were isolated 12 days later and homogenized (10% w/v) in phosphate-buffered saline. Aliquots were frozen and were subsequently used for infection. Mice received an inoculum of ∼1,000 spleen focus-forming units of FV by intravenous injection. Stocks of F-MLV-B and F-MLV-N helper viruses were grown in *Mus dunni* fibroblast cells. Mice received an inoculum of ∼10^4^ infectious units of F-MLV by intravenous injection. Neonatal F-MLV infection was performed by administering an inoculum of ∼4,000 infectious units of F-MLV-B to 1-day-old mice by intraperitoneal injection. All stocks were free of Sendai virus, Murine hepatitis virus, Parvoviruses 1 and 2, Reovirus 3, Theiler's murine encephalomyelitis virus, Murine rotavirus, Ectromelia virus, Murine cytomegalovirus, K virus, Polyomavirus, Hantaan virus, Murine norovirus, Lymphocytic choriomeningitis virus, Murine adenoviruses FL and K87, Mycoplasma sp. and LDV. For coinfection of FV and LDV, a similarly prepared stock of FV additionally containing LDV was also used^8^. For peptide immunization, mice received an intraperitoneal injection of a total of 12.5 nmol of synthetic env_124–138_ peptide mixed in Sigma Adjuvant System. FBL-3 tumour challenge was carried out by intravenous injection of 3 × 10^6^ FBL-3 cells. Ad5.pIX-gp70 stocks were prepared at a titre of 9 × 10^9^ viral genomes per ml by infection of 293A cells^16^. Approximately 5 × 10^8^ Ad5.pIX-gp70 viral genomes per mouse were administered intravenously.

### F-MLV copy number analysis

DNA copy numbers of F-MLV were determined by real-time quantitative PCR (qPCR) on DNA samples isolated from the spleen cell suspensions from infected mice, using primers specific to F-MLV *env* DNA (125 bp product): forward 5′-AAGTCTCCCCCCGCCTCTA-3′ and reverse 5′-AGTGCCTGGTAAGCTCCCTGT-3′. Signals were normalized for the amount of DNA used in the reactions based on amplification of the single-copy *Ifnar1* gene (150 bp product) with primers: forward 5′-AAGATGTGCTGTTCCCTTCCTCTGCTCTGA-3′ and reverse 5′-ATTATTAAAAGAAAAGACGAGGCGAAGTGG-3′. Copy numbers were calculated with a ΔΔC_T_ method and are expressed as copies per million cells.

### T-cell purification and adoptive transfer

Single-cell suspensions were prepared from the spleens and lymph nodes of donor CD45.1^+^ or CD45.2^+^ mice and CD4^+^ T cells were enriched using immunomagnetic positive selection (StemCell Technologies), at >96% purity. A total of 1 × 10^6^ CD4^+^ T cells were injected into CD45.1^+^CD45.2^+^ recipients via the tail vein. Where indicated, enriched EF4.1 CD4^+^ T cells were further purified (>98% purity) by cell sorting, performed on MoFlo cell sorters (Dako-Cytomation, Fort Collins, CO, USA).

### Bone marrow chimeras

Bone marrow cell suspensions were prepared by flushing the bone cavities of femurs and tibiae from donor mice with air-buffered Iscove's Modified Dulbecco's Media. MHC II-sufficient or -deficient bone marrow cells were injected separately into non-irradiated *Ighm*^−/−^ recipients. In this setting, only the missing lymphocyte population (B cells) is reconstituted^31^, creating B-cell-specific loss of MHC II. Each recipient received between 1 × 10^7^ and 3 × 10^7^ bone marrow cells. Mice were bled for assessment of reconstitution and were used for infection 8–10 weeks post bone marrow transfer.

### Flow cytometry

Single-cell suspensions were stained with directly conjugated antibodies to surface markers (Supplementary Table 1), obtained from eBiosciences (San Diego, CA, USA), CALTAG/Invitrogen, BD Biosciences (San Jose, CA, USA) or BioLegend (San Diego, CA, USA). Peptide-MHC II tetramers were prepared and used as previously described^16^. Multi-color cytometry were performed on Canto II, LSRFortessa X-20 (both from BD Biosciences) and CyAn (Dako) flow cytometers, and analysed with FlowJo v10 (Tree Star, Ashland, OR, USA) or Summit v4.3 (Dako) analysis software.

### Micropipette adhesion frequency assay

Two-dimensional TCR affinities were measured by a micropipette adhesion frequency assay as has been described in detail elsewhere^24^,^25^, using the T-cell hybridomas H5 and H18 and A^b^-env_125–135_ tetramer. Cells were brought into contact 50 times with the same contact time and area (*A*_c_), and an adhesion frequency (*P*_a_) was calculated. Surface pMHC and TCRβ densities were determined by flow cytometry and BD QuantiBRITE PE Beads for standardization (BD Biosciences). These parameters were then used to calculate two-dimensional affinity using the following equation: *A*_c_ × *K*_a_=−ln[1—*P*_a_(∞)] *m*_r_^−1 ^*m*_l_^−1^ where *m*_r_ and *m*_l_ represent TCR and pMHC surface densities, respectively.

### *In vitro* T-cell activation

Spleen or lymph node single-cell suspensions were prepared from EF4.1 or EVα3 mice and 5 × 10^5^ cells per well were stimulated in 96-well plates with the indicated amount of the index env_122–141_ epitope (DEPLTSLTPRCNTAWNRLKL), the shorter env_124–138_L epitope (PLTSLTPRCNTAWNR) or the altered peptides epitopes env_124–138_Y (PLTSYTPRCNTAWNR) and env_124–138_I (PLTSITPRCNTAWNR). Hybridoma cell lines were generated and stimulated as previously described^23^. T-cell activation was assessed 18 h later by flow cytometric detection of CD69 or CD44 (eBiosciences).

### Next-generation sequencing of the TCR repertoire

For TCR sequencing, naive or primed env-specific EF4.1 CD4^+^ T-cell subsets were purified by cell sorting (>98%) and RNA was isolated using the QIAcube (QIAGEN, Crawley, UK). Synthesis of cDNA was carried out with the High Capacity Reverse Transcription kit (Applied Biosystems, Carlsbad, CA, USA) with an added RNase-inhibitor (Promega Biosciences, Madison, WI, USA). A final clean-up was performed with the QIAquick PCR purification kit (Qiagen). Purified cDNA was then used as template for the amplification of *Trav14* (encoding Vα2)- or *Trav9* (encoding Vα3)-containing rearrangements, using the following primers: *Trav14* forward: 5′-CAAGCTTCAGTCTAGGAGGAATGGAC-3′; *Trav9* forward: 5′-CCAAGGCTCAGCCATGCTCCTGGC-3′; *Trac* common reverse: 5′-TAACTGGTACACAGCAGGTTCTGGG-3′. The forward *Trav*-specific primers were located in a promoter region common to all members of the respective *Trav* family and the common reverse was in the common constant region, thus amplifying the entire variable and joining coding region (∼500 bp product). Between 4,500 and 43,000 sequences per sample (450 bp median size) were obtained on a GS FLX System by GATC Biotech (Constance, Germany). Identification of productive rearrangements, *Trav* and *Traj* gene segment annotation and protein translation and CDR3 segment prediction were performed by the ImMunoGeneTics (IMGT) online tool HighV-QUEST (http://www.imgt.org)^67^.

### Immunohistochemistry

Frozen OCT (Dako)-embedded spleen sections were fixed in cold acetone, stained with directly conjugated monoclonal antibodies (Supplementary Table 1) against B220 (AlexaFluor 647, BD Biosciences) and IgD (PE, eBiosciences). Sections were also stained for the T- and B-cell activation antigen Ly77 (clone GL7, FITC, BD Biosciences) and FITC fluorescence was amplified with the AlexaFluor 488 signal amplification kit (Molecular Probes), according to manufacturer's instructions. Stained sections were mounted in fluorescent mounting medium (Dako) and viewed with a Leica TCS SP2 AOBS confocal microscope. The images were acquired with Leica confocal software using the 20 × or 40 × objective lenses.

### Statistical analyses

Statistical comparisons were made using SigmaPlot 12.0 (Systat Software, Germany). Parametric comparisons of normally distributed values that satisfied the variance criteria were made by unpaired Student's *t*-tests. Data that did not pass the variance test were compared with non-parametric two-tailed Mann–Whitney rank sum test. Comparison of Vβ family expression, hierarchical clustering and heat-map production was with Qlucore Omics Explorer (Qlucore, Lund, Sweden).

## Additional information

**Accession codes:** The Sequence Read Archive (SRA) and European Nucleotide Archive (ENA) accession numbers for the TCR sequences reported in this paper are SRX750575 and PRJEB11747, respectively.

**How to cite this article:** Merkenschlager, J. *et al.* Stepwise B-cell-dependent expansion of T helper clonotypes diversifies the T-cell response. *Nat. Commun.* 7:10281 doi: 10.1038/ncomms10281 (2016).

## Supplementary Material

Supplementary Supplementary Figures 1-5 and Supplementary Table 1
