# Supplementary material for: Stepwise B-cell-dependent expansion of T helper clonotypes diversifies the T-cell response
Source: Nat Commun. 2016 Jan 5;7:10281. doi: 10.1038/ncomms10281 (PMC4728444; doi:10.1038/ncomms10281)
Supplement: Supplementary — Figures 1-5 and Supplementary Table 1 [file ncomms10281-s1.pdf]

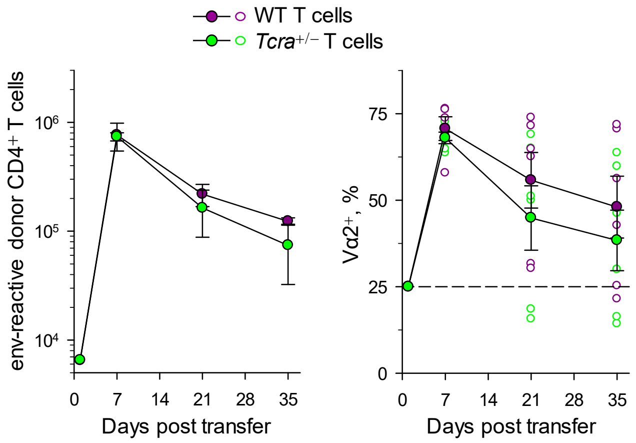

Supplementary Figure 1. Clonal composition of WT or *Tcra*$^{+/-}$ virus-specific CD4^{+} T cells. Absolute numbers (left) and Vα composition (right) of CD45.1^{+} WT or CD45.2^{+} *Tcra*$^{+/-}$ env-reactive donor EF4.1 CD4^{+} T cells in the spleens of CD45.1^{+}CD45.2^{+} WT recipients after cotransfer in equal numbers and FV infection (n=5-6 mice per time-point). Closed symbols are the means (±SEM); open symptoms are individual mice.

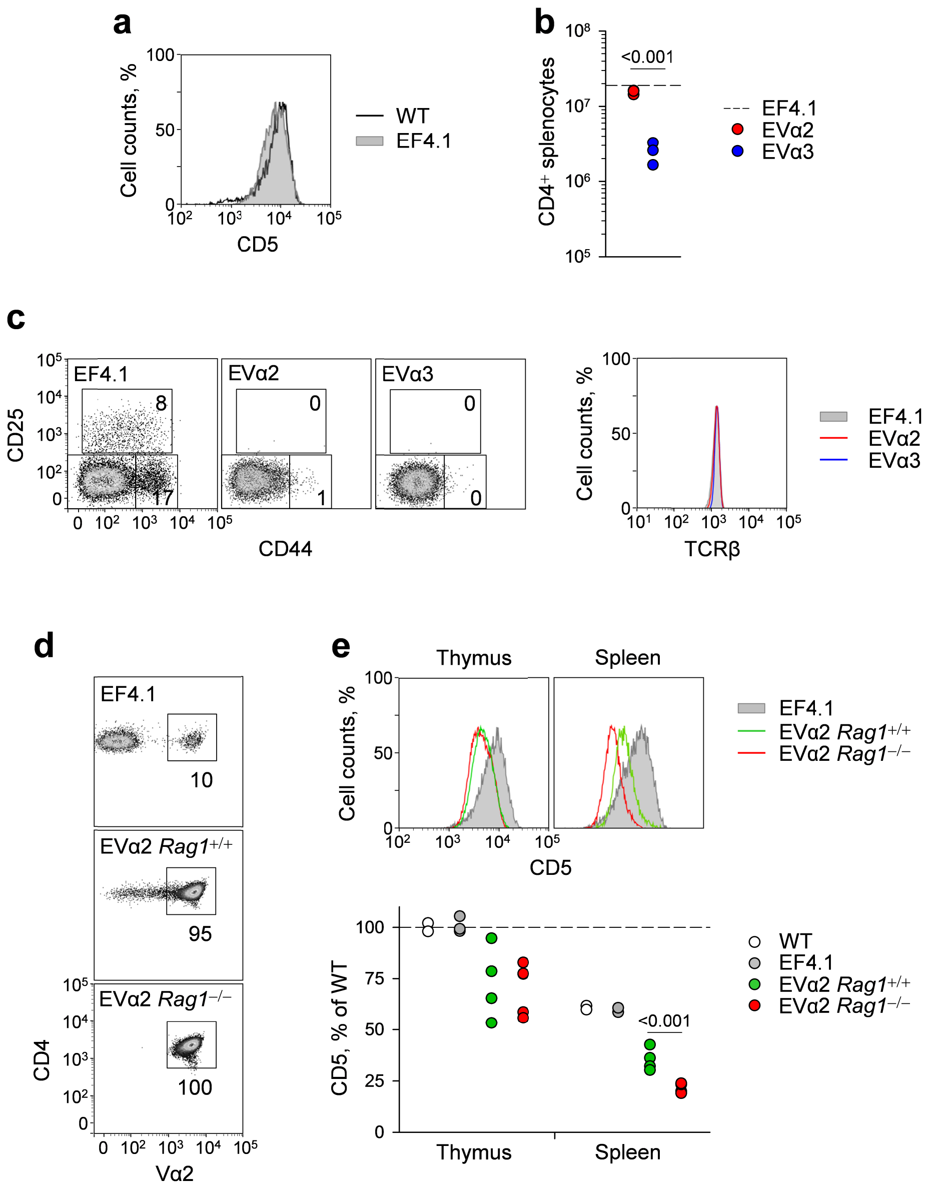

Supplementary Figure 2. T cell development, maintenance and phenotype in EF4.1, EVα2 and EVα3 mice. (**a**) CD5 levels in post-selection (CD4$^+$CD8$^-$) thymocytes from EF4.1 or WT mice. (**b**) Absolute number of splenic CD4$^+$ T cells from monoclonal (*Rag1*$^{-/-}$) EVα2 and EVα3 mice. (**c**) CD44, CD25 and TCRβ expression in the same cells as in (b). (**d**) Vα2 expression in CD4$^+$CD8$^-$TCRβ^{hi} thymocytes from EF4.1 mice or EVα2 mice either on a *Rag1*$^{+/+}$ (EVα2 *Rag1*$^{+/+}$) or *Rag1*$^{-/-}$ genetic background (EVα2 *Rag1*$^{-/-}$). (**e**) Flow cytometric examples of CD5 levels (top) and CD5 expression relative to WT levels (bottom) in post-selection (CD4$^+$CD8$^-$) thymocytes (Thymus) or splenic CD4$^+$ T cells (Spleen) from EF4.1, EVα2 *Rag1*$^{+/+}$ or EVα2 *Rag1*$^{-/-}$ mice. EVα2 *Rag1*$^{+/+}$ and EVα2 *Rag1*$^{-/-}$ T cells are gated as Vα2$^+$. CD5 levels in thymic and splenic T cells are expressed as percentage of CD5 levels in WT thymocytes. Each symbol represents an individual mouse.

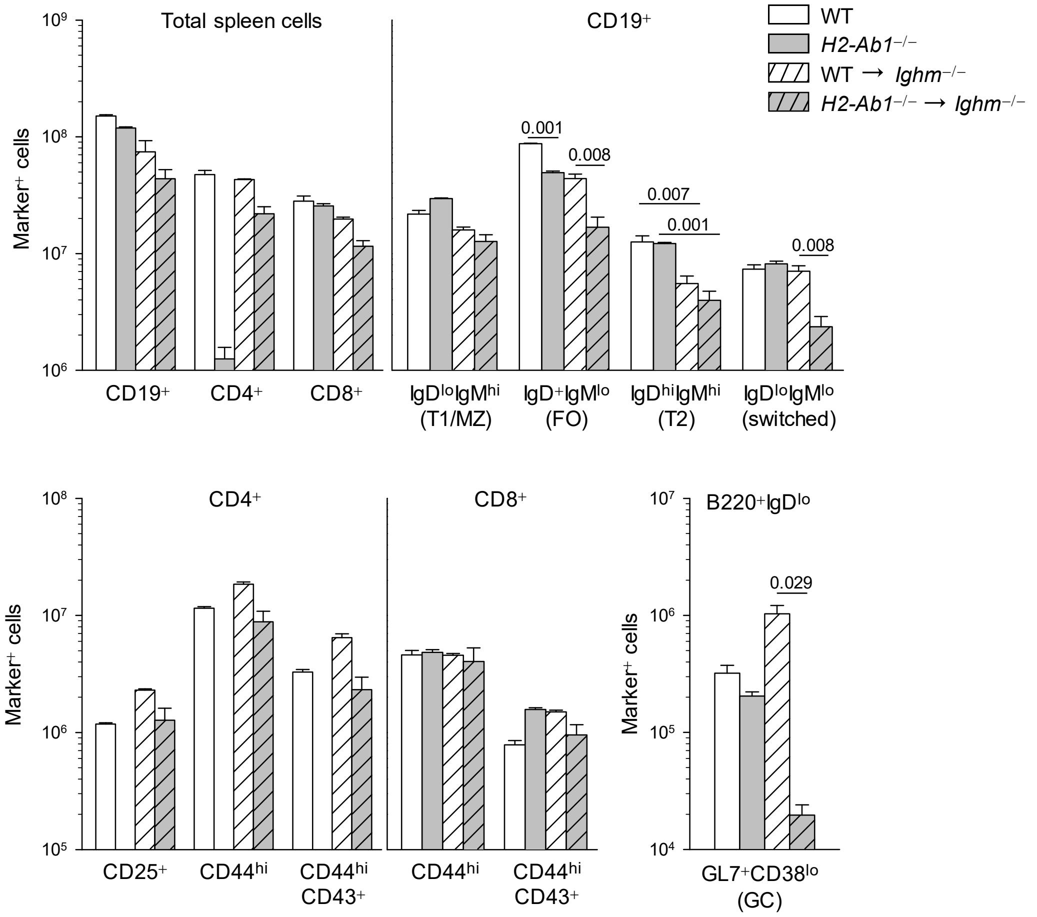

Supplementary Figure 3. Lymphocyte reconstitution in non-irradiated bone marrow chimeras. Total numbers of the indicated lymphocyte subsets in spleens of non-irradiated *Ighm*$^{-/-}$ recipients of MHC class II-sufficient (WT → *Ighm*$^{-/-}$) or -deficient bone marrow (*H2-Ab1*$^{-/-}$ → *Ighm*$^{-/-}$), 8 weeks and non-reconstituted WT and *H2-Ab1*$^{-/-}$ donor mice. Data are the means (±SEM) (n=3-4). T1/MZ, transitional 1/marginal zone; FO, follicular; T2, transitional 2; GC, germinal center.

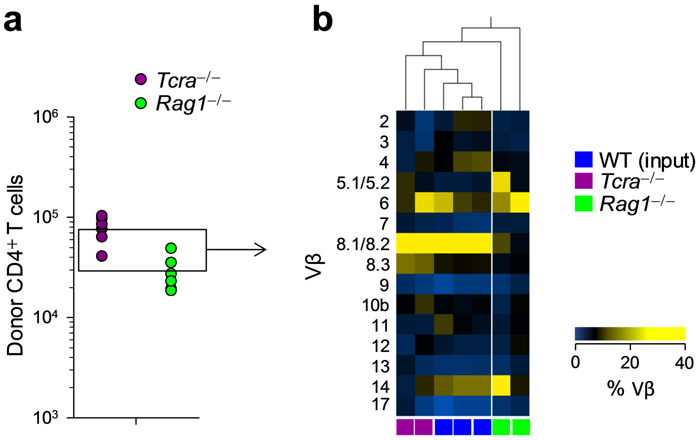

Supplementary Figure 4. TCR repertoire skewing in T cell-reconstituted *Rag1*$^{-/-}$ hosts independently of the extent of reconstitution. (**a**) Numbers of donor CD4^{+} T cells 21 days after reconstitution of *Tcra*$^{-/-}$ or *Rag1*$^{-/-}$ *Emv2*$^{-/-}$ recipients with purified WT CD4^{+} T cells. Each symbol is an individual mouse. Mice selected for comparable T cell reconstitution (rectangle) were then analyzed for Vβ usage. (**b**) Heat-map of hierarchically-clustered WT donors and *Tcra*$^{-/-}$ or *Rag1*$^{-/-}$ *Emv2*$^{-/-}$ recipients from (a), according to the frequency of the indicated Vβ family before (WT (input)) or 21 days after T cell reconstitution (*Tcra*$^{-/-}$ or *Rag1*$^{-/-}$). Each column is an individual mouse.

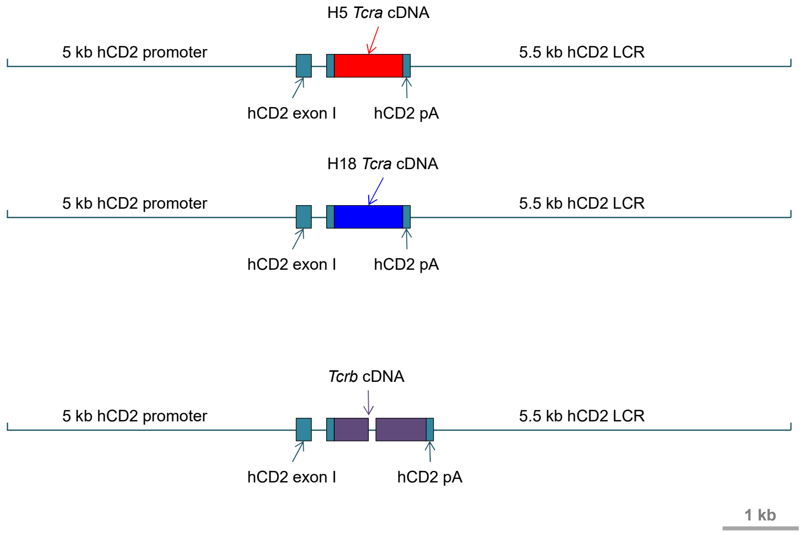

Supplementary Figure 5. TCR constructs used for the generation of EVα2 and EVα3 mice. The H5 and H18 env-specific $CD4^+$ T cell clones from the polyclonal repertoire of EF4.1 mice were used for cloning of the *Tcra* cDNAs used to create EVα2 and EVα3 mice, respectively. Each of these were cloned into the hCD2-VA expression cassette, which encompasses a 5 kb human CD2 promoter region, the first exon (with mutated ATG) and intron of the human CD2 gene and a 5.5 kb locus control region, at the 3' end of the gene, which is necessary for appropriate expression in T cells. A similar construct with the Tcrb cDNA from the original EF4.1 mice was also used (bottom). *Tcra* and *Tcrb* constructs were co-injected to produce founder mice, genotyped by Transnetyx (Cordova, TN 38016, United States). Correct expression and function of the transgenes was verified by data shown in Fig. 2e and Supplementary Fig. 2.

Supplementary Table 1. Antibodies used in this study.

Antibody	Vendor	Clone	Catalogue no.	Dilution
CD25 APC	eBioscience	pc61.5	17-0251-82	1/200
CD38 APC	eBioscience	90	17-0381-82	1/200
CD4 APC	eBioscience	GK1.5	12-0041-82	1/200
CD4 FITC	Insight Biotech	RM4-5	11-0042-82	1/200
CD4 PE	eBioscience	GK1.5	12-0041-85	1/300
CD4 PE-Cy7	eBioscience	GK1.5	25-0041-82	1/200
CD43 PE	BD Biosciences	1B11	558762	1/500
CD44 Pacific Blue	Biolegend	IM7	103020	1/200
CD44 V500	BD Biosciences	IM7	560780	1/200
CD45.1 eFluor® 450	eBioscience	A20	48-0453-82	1/200
CD45.1 PE	eBioscience	A20	12-0453-83	1/200
CD45.2 APC-Cy7	Biolegend	104	109824	1/200
CD45.2 APC-eFluor® 780	eBioscience	104	47-0454-82	1/200
CD45R (B220) PE-TR	Invitrogen	RA3-6B2	RM2617	1/300
CD5 PE	eBioscience	53-7.3	12-0051-83	1/1000
CD69 FITC	eBioscience	H1.2F3	11-0691-85	1/200
CD8a FITC	eBioscience	53-6.7	11-0081-85	1/600
GL7 FITC	BD Biosciences	GL7	553666	1/200
IgD PE	eBioscience	11-26c	557359	1/200
Ly-6C Biotin	eBioscience	AL-21	557359	1/300
MHC Class II (I-A/I-E) FITC	eBioscience	M5/114.15.2	11-5321-85	1/300
TCR beta APC-eFluor® 780	eBioscience	H57-597	47-5961-82	1/200
TCRb APC	eBioscience	H57-597	17-5961-81	1/200
TCRb APC-Cy7	eBioscience	H57-597	10-5961-82	1/200
Va 3.2 FITC	eBioscience	RR3-16	553219	1/200
Va 3.2b,c TCR FITC	BD Biosciences	RR3-16	553219	1/200
Va2 TCR FITC	BD Biosciences	B20.1	553288	1/200
Vb TCR Screening Panel	BD Biosciences	Vb various	557004	1/300
Vα2 TCR APC	eBioscience	b20.1	17-5812-82	1/200
